# Dual-pathway protection: physical activity buffers the relationship between perceived stress and problematic smartphone use via social anxiety by a moderated mediation model in Chinese college students

**DOI:** 10.3389/fpubh.2025.1681556

**Published:** 2025-10-30

**Authors:** Ningning Liu, Xiaoyuan Han, Xinghong Dai, Guofeng Zhang

**Affiliations:** ^1^College of Basic Medical Sciences, Jining Medical University, Jining, China; ^2^Department of Psychology, Chengde Medical University, Chengde, Hebei, China; ^3^School of Physical Education, Hunan University, Changsha, China; ^4^Basic Department, Jining Polytechnic, Jining, China

**Keywords:** physical activity, problematic smartphone use, college students, social anxiety, perceived stress, a moderated mediation model

## Abstract

**Objective:**

This study investigates the dual-pathway mechanism through which physical activity mitigates problematic smartphone use (PSU) among Chinese college students, examining the mediating role of social anxiety and the moderating effects of physical activity within a stress-PSU framework.

**Methods:**

General stress theory was introduced to explore the intrinsic mechanisms by which perceived stress influences PSU. A total of 2,905 undergraduate students from universities in Shandong Province were selected using a convenient sampling method. Validated scales were used to measure perceived stress (PSS-14), social anxiety (IAS), physical activity (PARS-3) and PSU tendency (MPATS). Data analysis included correlation tests, regression analysis and moderated mediation modeling (PROCESS Macro Model 4/15).

**Results:**

(1) There was a significant positive correlation between perceived stress, social anxiety and problematic smartphone use (All *P* < 0.01). Significant gender differences were found in social anxiety (*P* < 0.001) and problematic smartphone use (*P* < 0.05). (2) Path analysis revealed: A significant direct effect of perceived stress on PSU (β = 0.537, 95%*CI*[0.490, 0.586]), accounting for 70.94% of the total effect. Social anxiety mediated 29.06% of the effect (indirect effect = 0.220, 95%*CI*[0.195, 0.244]). (3) Physical activity demonstrated dual moderating effects: Buffered the direct stress-PSU pathway (β = −0.005, *P* < 0.001). Attenuated the social anxiety-PSU association (interaction β = 0.002, *P* < 0.05)

**Conclusion:**

Physical activity disrupts the stress-PSU cycle through two synergistic mechanisms: directly reducing stress-driven addiction and decoupling social anxiety from excessive smartphone use. Universities should implement structured physical activity programs as a dual-path intervention against PSU.

## 1 Introduction

The widespread adoption of smartphones has significantly transformed lifestyles in the digital era, where “everything is connected,” and mobility has increasingly become the norm (1). As a prominent emerging force in the digital age, college students have experienced a substantial rise in smartphone ownership and usage, driven by advancements in mobile internet technology, the proliferation of social media, and the diversification of entertainment and recreational activities (2). In China, according to the 53rd Statistical Report of the China Internet Network Information Center (CNNIC), 19.8% of the country's internet users are college students or above. While smartphones offer significant convenience for students' academic and daily activities, they also function as a “double-edged sword,” as excessive use can lead to behavioral addiction (3, 4), commonly referred to as problematic smartphone use (PSU). PSU is associated with various physical symptoms, including dizziness, chest tightness, palpitations, and digestive dysfunction (5, 6), as well as psychological issues such as social withdrawal, anxiety, depression, and stress, which negatively impact students' physical and mental wellbeing (7). Several studies (8, 9) have reported that the prevalence of PSU among Chinese college students ranges from 63% to 86%, with an increasing proportion of moderate to severe cases. This growing trend has become a major social concern, highlighting the urgent need to investigate the underlying mechanisms of PSU and develop effective interventions.

Problematic smartphone use is a form of addictive behavior characterized by excessive smartphone engagement that significantly impairs mental health (10, 11).Previous studies have examined the factors that influence PSU, several studies have confirmed that perceived stress is a precursor to PSU (12–14). It has been demonstrated that stress-induced cognitive and behavioral dysregulation contributes to behavioral addictions in schizophrenia. Research indicates that disorganized thinking, a core symptom of schizophrenia (15), arises through a mechanism closely linked to stress-induced depletion of cognitive resources, alteration of intestinal flora amplifies this process through metabolic regulation (16). This indicates that stress triggers behavioral problems through emotional and cognitive mediators. Accumulated stress and a lack of coping strategies significantly increase the risk of PSU (17, 18). While high stress exposure directly or indirectly affects PSU by reinforcing perceived stress. Research indicates that perceived stress can affect PSU directly and indirectly through certain mediating variables. Seo et al. (19) conducted a longitudinal study on smartphone addiction that suggests anxiety is a reason people use their phones for extended periods. Social anxiety, as a specific form of anxiety, has been confirmed as a key risk factor for exacerbating PSU. Previous studies have examined the factors that influence PSU (20, 21). Elhai et al. (22) also noted that individuals with high perceived stress often experience negative emotions and have difficulty interacting with others (23, 24).which exacerbates social anxiety and ultimately reinforces PSU behavior, This forms a potential pathway of “perceived stress-social anxiety-PSU”.

Although existing studies have explored the relationship between perceived stress, social anxiety, and PSU, most studies adopt a negative perspective and lack in-depth exploration of positive intervention strategies. Physical activity is a key protective factor for PSU. Its mechanism of action must be understood within a more systematic theoretical framework. According to the cognitive-behavioral model of social anxiety (25), individuals with high social anxiety tend to avoid real-life social interactions and instead rely on online interactions, forming a behavioral reinforcement cycle. And physical activity can break this cycle through a dual path: on the one hand, it directly relieves stress (26), and on the other hand, it weakens the association between social anxiety and PSU by providing alternative social situations (27): The moderating effect of physical activity on the “social anxiety → PSU” pathway can be explained by the social substitution hypothesis. Physical activity provides opportunities for face-to-face interaction, which reduces reliance on virtual socializing (28). Group exercise also enhances social self-efficacy (27). These factors weaken the conversion of social anxiety into PSU. However, current research has not tested the moderating effect of physical activity on the mediating pathway of social anxiety in the stress-PSU model, nor has it validated the moderating mechanism for the latter half of the pathway (social anxiety → PSU). Furthermore, a growing body of research has documented the protective role of physical activity against PSU, highlighting its potential to mitigate negative emotional states and improve self-control, which are crucial mechanisms in reducing addictive behaviors (29, 30).

Moreover, most research has been conducted from a “problem-oriented” perspective, lacking sufficient theoretical support, thereby limiting the depth of mechanism-based investigations. Research predominantly focuses on adolescents, with insufficient attention given to university students as a distinct population. Compared to adolescents, university students are in a critical transitional phase of life, characterized by greater regularity, autonomy, and social coordination. Investigating the causes and underlying mechanisms of PSU among university students is essential for developing targeted and effective prevention and intervention strategies, providing valuable scientific insights. Therefore, examining PSU in university students is particularly significant and holds unique academic value.

In this study, we innovatively incorporate physical activity as a positive variable, aiming to address gaps in existing research on the relationship between perceived stress and problematic smartphone use. Through this research, we seek to enhance a comprehensive understanding of this complex phenomenon. The study adopts the General Strain Theory as an analytical framework (31), which provides a logical chain linking stressors, negative emotions, and problematic behaviors, offering a solid theoretical foundation for investigating problematic smartphone use. Targeting university students as the research subjects, this study approaches the issue from the perspectives of positive psychology and sports psychology to explore the underlying causes and internal mechanisms of problematic smartphone use. We hope that this research will draw widespread societal attention to the phenomenon of problematic smartphone use, encouraging university students to actively engage in physical activity as an effective means to overcome and mitigate problematic smartphone use, thereby better coping with the psychological challenges posed by the digital era.

**Figure 1:**
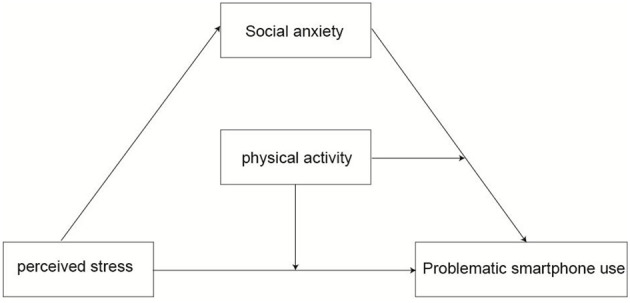
Hypothetical variable model. *Note* Independent variable (**X**): perceived stress; Dependent variable (**Y**): Problematic smartphone use; Mediating variable (**M**): Social anxiety; Moderating variables (**W**): physical activity.

This study takes the general strain theory as the analytical framework (31). This theory was first proposed by the American scholar Robert Agnew in the article “The Foundations of Crime”. Its core viewpoint can be summarized as the causal chain of “stressors—negative emotions—problem behaviors”, that is, stress can cause individuals to have negative emotions, and then trigger problem behaviors. Based on this theory, this study introduces perceived stress as the independent variable, problematic smartphone use as the dependent variable, social anxiety as the mediating variable, and physical activity as the moderating variable, attempting to deeply explore the internal mechanism by which perceived stress affects problematic smartphone use. Based on this, the following research hypotheses are proposed:

H1: Perceived stress has a significant positive predictive effect on college students' problematic smartphone usage; H2: Social anxiety plays a mediating role between perceived stress and problematic smartphone use; H3: Physical activity plays a regulatory role in relationships. If college students frequently engage in physical activity, the mediating effect of social anxiety will be more significant. The research variable model is shown in Figure 1.

## 2 Methods and materials

### 2.1 Participants and data collection

From May to June 2024, we adopted diversified recruitment channels, including promotion through social media such as student forums, Weibo, WeChat, QQ Space, and in the form of campus posters. We recruited 3,216 undergraduate students from 5 universities in Shandong Province to participate in the survey. Participants accessed the online questionnaire on the Questionnaire Star platform (https://www.wjx.cn/). The survey was launched by scanning the code to answer the questions, and the system was set up to a mode in which an IP address could only be submitted once to avoid the same respondent answering the questions multiple times. Two psychology students who had received professional training were selected as the chief examiners of this survey. Participation was voluntary and completed the questions anonymously. The answering time was about 10 min. Before distributing the questionnaire, Participants would read standardized instructions explaining the research purpose, methods, and significance. During the survey, detailed guidance was provided for each question to improve response quality. After collecting the questionnaires, the chief examiners promptly checked them to ensure completeness and standardization, discarding any non-standard responses to retain only the valid questionnaires.

Out of the 3,216 questionnaires distributed, 311 were discarded due to incomplete answers or duplication, and 2,905 valid questionnaires were finally collected with an effective response rate of 90.3%. The study subjects were aged between 18 and 22 years [(19.31 ± 0.95) years]. Among them, 1,181 (40.65%) were male and 1,724 (59.35%) were female. In this study, the R language semPower package was used to perform an a priori analysis to calculate the minimum number of samples (32). The calculated degrees of freedom were 1,190, and the minimum sample size must be 30 or more. Our final sample (*N* = 2,905) exceeded this threshold, ensuring adequate statistical power. The study was approved by the Ethics Committee of Jining Medical University (JNMC-YX-2024-076), and all participants signed informed consent forms.

### 2.2 Measures

#### 2.2.1 Demographic information

We accessed general demographic information of participants in the beginning of the questionnaire, including age, gender, major, grade, place of origin, the number of siblings, family status.

#### 2.2.2 Mobile phone addiction tendency scale (MPATS) for college students

Students' problematic smartphone use is the dependent variable in this paper. The MPATS scale (33), developed by Jie Xiong, Zongkui Zhou, and colleagues, was utilized to assess university students' tendencies toward smartphone addiction. Including 16 questions such as “Do I often let my mobile phone get in the way of other important things?” This scale consists of 16 items categorized into four dimensions: withdrawal symptoms, prominent behaviors, social consolation, and mood changes. Each item is rated on a scale of 1–5, resulting in a total score ranging from 16 to 80, with higher scores indicating a greater tendency toward smartphone addiction. It is widely used in research in psychology, education and other related fields. In this study, the Cronbach's α coefficient for the scale was 0.928, which indicates that the scale has a good level of reliability.

#### 2.2.3 Perceived Stress Scale (PSS-14)

The independent variable used in this study was perceived stress. The perceived Stress Scale was introduced by Cohenet al. (34) and is designed to quantify, through a series of questions, an individual's feelings of stress when experiencing specific events or demands of the life situation. In this study, the Chinese version of the perceived Stress Scale translated by Crockett et al. was used, which includes questions such as ‘In the past month, have you often felt that you had no control over important things that happened in your life? with 14 items scored on a Likert 5-point scale, with a minimum score of 1 (no stress) and a maximum score of 5 (severe stress), and a total score ranging from 0 to 56. Scores that below the 25th percentile (0–17) indicate low stress, 25–75th percentile (18–28.5) indicates moderate stress, and above the 75th percentile (28.6–56) indicates high stress. The scale has stable psychometric properties and has been used in a wide range of studies with different populations. The Cronbach's alpha coefficient in this study was 0.87, which indicates that the scale has a good level of reliability.

#### 2.2.4 Interaction Anxiousness Scale (IAS)

The mediating variable is social anxiety. The Interaction Anxiousness Scale was originally developed by Leary in 1983(35) and is mainly used to assess the tendency of subjective social anxiety experiences that are independent of behaviors. The severity of social anxiety among university students was assessed using the IAS scale (36) revised by Chunzi Peng et al. Including questions such as “Do I often feel nervous and uncomfortable in social situations?” and so on. This scale includes 15 items, 11 of which were rated positively and 4 negatively (questions 3, 6, 10, 15). Each of these items was scored using Likert 5-point scoring method, with the lowest score being 1 point (completely inconsistent) and the highest score being 5 points (completely consistent). The scores for the reverse-scored questions are calculated in reverse order before being added to the total score. The scores for all items are added together to give a total score ranging from 15 to 75 points. The higher the score, the higher the severity of individual's social anxiety. The Cronbach's α coefficient of the scale in this study was 0.852, which indicates that the scale has a good level of reliability.

#### 2.2.5 Physical Activity Rating Scale (PARS-3)

Physical activity is the moderating variable in this paper. The Physical Activity Rating Scale (PARS-3) (37), originally developed by the Japanese scholar Kimio Hashimoto and later revised by Chinese scholar Deqing Liang and colleagues, is used to assess the physical activity levels of college students.The scale contains 4 questions and 3 dimensions, namely, activity intensity, activity time and activity frequency. Including “In the past week, on how many days did you get at least 30 min of vigorous physical activity?” And so on. Each question is scored on a 5-point Likert scale, with a minimum score of 1 and a maximum score of 5 for the activity intensity and frequency questions, and frequency.and a minimum score of 0 and a maximum score of 4 for the activity duration question. The total score is the product of activity intensity, activity time and activity frequency. The higher the score, the greater the amount of activity: less than 19 points indicates small amount of activity, 20–42 points indicate medium amount of activity, and more than 42 points indicates the activity duration questions. The test-retest reliability of the scale is 0.82, which indicates that the scale has a good level of reliability.

### 2.3 Statistical analysis

First, we use SPSS 26.0 to conduct common method bias test, and scale reliability analysis. We also conduct analysis including descriptive statistics, independent samples *t*-test, correlation analysis, and explore the relationship between problematic smartphone use, perceived stress and social anxiety using Pearson correlation analysis. Secondly, mediation analysis was conducted using Model 4 in the SPSS process 3.3 plug-in prepared by Hayes (65), and mediation effect tests were conducted using the bootstrap (5,000 times) method to explore the mediating role of social anxiety and mediation effect test was conducted using Model 15 to examine whether the mediating effect of social anxiety was moderated by physical activity.

## 3 Results

### 3.1 Common method bias test

Given the self-report nature of the questionnaires, common method bias may exist. This study used procedural controls and Harman's single-factor test. Before the test, anonymity and reverse scoring were implemented. Harman's test showed that the variance explained rate of the largest common factor was 30.81%, below the 40% threshold, indicating no serious common method bias.

### 3.2 Descriptive statistics and correlation analysis

Table 1 presents the means, standard deviations, and correlation coefficients of the variables. The correlation coefficient between social anxiety and problematic smartphone use was 0.514 (*P* < 0.01), and the correlation coefficient between perceived stress and problematic smartphone use was 0.500 (*P* < 0.01), the correlation coefficient between perceived stress and social anxiety was 0.390 (*P* < 0.01), indicating a significant positive correlation between the two variables. These preliminary analyses support the subsequent hypothesis testing.

**Table 1:** Correlation analysis of variables.

**Variate**	**M±SD**	**Perceived stress**	**Social anxiety**	**Problematic smartphone use**	**Physical activity**
Perceived stress	41.58 ± 8.20	1			
Social anxiety	46.48 ± 9.20	0.390^**^	1		
Problematic smartphone use	42.84 ± 12.49	0.500^**^	0.514^**^	1	
Physical activity	8.42 ± 2.28				1

^**^P < 0.01.

### 3.3 The mediating role of social anxiety between perceived stress and problematic smartphone use among college students and the moderating role of physical activity

The sequential test method was used for regression analysis, and the percentile Bootstrap method with bias correction was used to calculate the 95% confidence interval of the mediating effect. To investigate the possible correlation and mediating effect of perceived stress, problematic smartphone use, social anxiety and physical activity. The steps are as follows: all the measured variables are standardized. According to Hayes' suggestion, Model 4 in the SPSS macro program Process 3.3 was used to verify the mediating role of social anxiety between perceived stress and problematic smartphone use, and Model 15 tested whether the mediating effect of social anxiety was moderated by physical activity.

**Figure 2:**
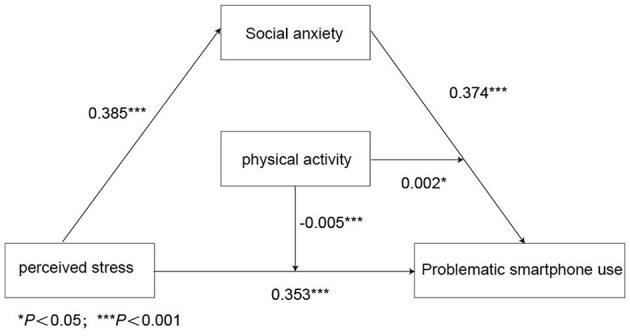
The mediating effect model of social anxiety between perceived stress and problematic smartphone use. *Note* Physical activity negatively predicted problematic smartphone use. The interaction between physical activity and social anxiety significantly positively predicted problematic smartphone use of college students (β = 0.002, *P* < 0.05), that is, physical activity could regulate the latter half of the mediating effect of social anxiety between perceived stress and problematic smartphone use. Physical activity showed a significant negative correlation between perceived stress and problematic smartphone use (β = −0.005, *P* < 0.001), that is, physical activity could regulate the latter half of the path between perceived stress and problematic smartphone use.

See Tables 2–4 and Figures 1, 2. In the model, perceived stress was the independent variable, and the problematic smartphone use was the dependent variable. The social anxiety and physical activity were the intermediate variables, and indirect effects were tested. The results showed that perceived stress positively predicted problematic smartphone use (β = 0.353, *P* < 0.001), and the correlation between the two variables was still significant when the mediating variable social anxiety was included (β = 0.374, *P* < 0.001). Social anxiety had a partial mediating effect between the perceived stress and problematic smartphone use of college students, with an effect value of 0.220. The 95% confidence interval is (0.195–0.244), accounting for 29.06% of the total effect.

**Table 2:** Mediation effect of social anxiety.

**Variate**	**Social anxiety**		**Problematic smartphone use**	
	β	***t***	β	***t***
Gender	0.155	9.221^***^	0.008	0.509
Grade	0.013	0.755	0.037	2.503^*^
Perceived Stress	0.385	22.847^***^	0.353	22.047^***^
Social Anxiety			0.374	23.059^***^
*R2*	0.177	0.371		
*F*	207.332^***^	428.090^***^		

^*^P < 0.05, ^***^P < 0.001.

**Table 3:** Bootstrap analysis of the mediating effects of social anxiety on perceived stress and problematic smartphone use.

**Pathway**	**Effect value**	**SE**	**LLCI**	**ULCL**	**Effect ratio (%)**
Total effect	0.757	0.024	0.709	0.805	100.00
Direct effect	0.537	0.024	0.490	0.586	70.94
Indirect effect	0.220	0.013	0.195	0.244	29.06

**Table 4:** Moderating effect of physical activity.

**Dependent variable**	**Independent variable**	**β**	***SE***	***t***	***P***	**95%*CI***
Problematic smartphone use	Gender	−0.124	0.387	−0.321	0.748	−0.883 to 0.634
	Grade	0.825	0.372	2.221	0.026	0.097 to 1.554
	Perceived stress	0.657	0.035	18.741	< 0.001	0.588 to 0.726
	Social anxiety	0.443	0.034	13.192	< 0.001	0.377 to 0.509
	Physical activity	0.055	0.051	1.071	0.284	−0.046 to 0.155
	Perceived stress × physical activity	−0.005	0.001	−4.503	< 0.001	−0.007 to −0.003
	Social anxiety × physical activity	0.002	0.001	2.094	0.036	0.001 to 0.004

Model 15 in the PROCESS macro was used to examine whether physical activity moderates the latter half of the mediating effect of social anxiety between perceived stress and problematic smartphone use. The results showed (Table 4) that the interaction between physical activity and social anxiety significantly positively predicted problematic smartphone use among college students (β = 0.002, *P* < 0.05). Physical activity showed a significant negative correlation between perceived stress and problematic smartphone use (β = −0.005, *P* < 0.001). Therefore, physical activity could regulate the latter half of the path between perceived stress and problematic smartphone use.

## 4 Discussion

The university stage is a critical stage for the formation and stable development of concepts and behaviors, and it is important to explore the emergence mechanism of problematic smartphone use at university, and to provide appropriate guidance and effective intervention for their healthy development. Perceived stress and problematic smartphone use among college students are closely related, however, existing studies have rarely explored the effects of perceived stress on problematic smartphone use among college students (38, 39). This study examined the effects of perceived stress on college students' problematic smartphone use and the mediating mechanisms of social anxiety and the potential moderating mechanisms of physical activity from the perspective of general stress theory. This study found that perceived stress had a significant positive predictive effect on college students' problematic smartphone use, and the results verified Hypothesis 1. It is worth noting that the direct predictive effect of perceived stress on college students' problematic smartphone use was strengthened by the addition of the mediator variable of social anxiety, and physical activity played a potential moderating role in the triple relationship.

### 4.1 The influence of perceived stress on problematic smartphone use in college students

The results of this study show that perceived stress is a risk factor for problematic smartphone use and positively predicts such behavior among college students (40, 41) which confirmed Hypothesis 1.In other words, the higher the perceived stress, the greater the likelihood of engaging in problematic smartphone use (42), which is consistent with previous studies (40, 43, 44). This conclusion supports the General Strain Theory, which suggests that problematic behaviors are primarily driven by various forms of pressure or stressful events (31). Under stress, individuals tend to adopt different coping mechanisms to alleviate the negative emotions caused by the pressure. This theory has been effectively applied to explain addictive behaviors, such as how high-stress experiences increase an individual's desire for substances, potentially leading to addiction (45). The stress coping model of addiction suggests that when an individual experiences high pressure, they are likely to become addicted to their mobile phone in order to release emotions, relieve stress and escape from reality through excessive smartphone use, which is a negative stress coping style (46, 47). Furthermore, the intricate connections between stress, PSU, and broader wellbeing are increasingly recognized. Research by Wang et al. (48) revealed that stress and smartphone addiction served as chain mediators linking physical activity to sleep quality, underscoring the necessity for future studies to develop more comprehensive models that incorporate a wider network of variables to fully elucidate the underlying mechanisms.

Additionally, from the perspective of self-control theory, when an individual experiences high levels of stress, their self-control resources are depleted, potentially leading to pathological behaviors. The greater the perceived stress, the lower an individual's level of self-control, which explains the tendency to seek instant gratification (such as problematic smartphone use) as a means of stress relief. Problematic smartphone use, as a form of behavioral addiction, offers vicarious satisfaction and may serve as a coping mechanism for managing stress or negative emotions (14). Research has shown that college students under high stress are more likely to turn to smartphones as a way to alleviate pressure (49).

As an important aspect of positive psychological qualities, perceived stress can influence and reshape students' cognition and behavior. To address this, educators can provide professional psychological counseling, enhance students' coping abilities, and reduce their perceived stress levels. Universities can also organize activities aimed at developing problem-solving skills and offer diverse outlets for emotional expression.

### 4.2 The mediating role of social anxiety in the relationship between perceived stress and problematic smartphone use

Davis's “cognitive-behavioral” model points out that social anxiety is one of the important components of the psychopathology of addictive behaviors (50). The results of this study show that perceived stress influence problematic smartphone use among college students through the mediating role of social anxiety. Hypothesis 2 was confirmed. On the one hand, perceived stress positively predicts social anxiety levels in college students; that is, higher perceived stress are associated with increased levels of social anxiety, which aligns with the findings of Borchers et al. (51). This study pointed out that the higher the perceived stress of migrant children, the more negative the psychological expectation of social situation, which leads to higher social anxiety experiences (52). Meanwhile, social anxiety may be one of the precursor factors for the formation of problematic smartphone use (49). Social anxiety positively predicts problematic smartphone use among college students; specifically, higher levels of social anxiety correspond to more pronounced problematic smartphone use, which aligns with the findings of Korniienko et al. (53). The cognitive-behavioral model of social anxiety shows that individuals with social anxiety are more likely to avoid social behavior through social media, which leads to problematic smartphone use (54). In conclusion, reducing social anxiety is a key factor in problematic smartphone use among students growing up under high stress.

High levels of social anxiety often rely on the social features of smartphones as a substitute for face-to-face interactions, which can result in poorer-quality interpersonal relationships (55, 56). Colleges can provide group counseling focused on social skills training to help students with interpersonal difficulties develop healthy relationships. In addition, studies have proven that physical activity, by diverting attention from prolonged Smartphone use, not only strengthens the body, but also enhances peer relationships, relieves study and life pressures, expands interpersonal relationships, expand the scope of interpersonal communication and improves interpersonal communication skills, which is an important way to effectively control and correct problematic smartphone use. Regular participation in physical activity, especially group activity, is conducive to obtaining social support, promoting the development of social skills, and establishing good interpersonal relationships, which can prevent and alleviate the occurrence of problematic smartphone use among college students.

### 4.3 The moderating role of physical activity

Our findings are consistent with existing domestic research: Physical activities can not only regulate negative emotions by enhancing psychological resilience (57) but also alleviate mobile phone dependence by improving self-control ability (29, 58). The synergistic effect of these internal protective factors jointly constitutes its psychological mechanism for buffering stress. The results of this study showed that physical activity had a significant negative moderating effect on the mediating effect of social anxiety in the influence of perceived stress on problematic smartphone use. In other words, when perceived stress was added as an independent variable, problematic smartphone use as a dependent variable, and social anxiety as a mediator, the predictive effect of perceived stress on problematic smartphone use changed with the addition of the moderating variable of physical activity, supporting hypothesis 3. Magalhaes et al. (26) found significant reduction in perceived stress after aerobic, physical, joint and activity groups in experiments, which is consistent with the present study. Problematic smartphone use is a behavioral addiction (27), which can be explained by the fact that activity affects an individual's addictive state by releasing dopamine signaling capacity and increasing self-efficacy (24, 59), and that the positive predictive effect of perceived stress on problematic smartphone use among college students decreases with increasing physical activity, thus reducing the incidence of problematic smartphone use among college students.

In addition, the results of the study showed that physical activity has a positive moderating effect on the relationship between social anxiety and problematic smartphone use among college students. College students who regularly participate in sports activities usually possess strong mental toughness and perseverance, which is sufficient to resist the intrusion of social anxiety triggers from internal and external sources, and to prevent and alleviate the occurrence of social anxiety. Physical activity serves as an effective alternative for mitigating social anxiety and subsequent PSU, primarily by offering real-world social opportunities that enhance peer relationships and build psychological capital, thereby reducing reliance on virtual social interaction (60). Another study supplemented this mechanism from the perspective of subjective wellbeing, finding that physical activity among college students during the COVID-19 period could indirectly reduce the risk of Internet addiction by enhancing subjective wellbeing, and the mediating effect of subjective wellbeing accounted for 72.81% of the total effect. This provides a new explanatory dimension for the psychological path by which exercise improves addictive behavior (61). This moderating mechanism is further corroborated by its protective role across different psychological pathways—such as alleviating loneliness (62)—highlighting the broad buffering capacity of physical activity in the development of behavioral addictions. Based on this, physical activity is a key variable in ameliorating problematic smartphone use among college students. Appropriate physical activity, such as running, swimming, and fitness training, can reduce the frequency of mobile phone use and enhance self-efficacy, which is an effective way to improve individuals' problematic smartphone use (63). Further randomized controlled trials confirmed that acute moderate-intensity aerobic exercise (such as 30 min of treadmill exercise with heart rate maintained at 45–68% heart rate reserve) could significantly reduce the mobile phone cravings of college students with mobile phone dependence, providing direct evidence for the immediate intervention effect of the above-mentioned exercise types (64).Colleges and universities should organize more group physical activities, enhance social confidence actively integrate into the collective environment, guide students to put down their mobile phones and head to the playground, thus creating a united and friendly social environment through physical activities, in this way, it can thereby relieve college students' pressure and enhance their health literacy.

### 4.4 Implications

The main theoretical advancement of this study lies in establishing physical activity as a positive moderating variable in the general stress theory, revealing the mechanism by which it breaks the “stress-problematic mobile phone use” cycle through dual pathways: it not only directly alleviates the negative impact of stress on adults but also weakens the association between social anxiety and problematic mobile phone use.

Based on the above findings, universities can incorporate structured physical activities into the overall intervention system for problematic mobile phone use at the practical level. Specifically, a variety of compulsory physical education courses can be implemented throughout the school, and team sports and sports clubs can be vigorously developed to enhance students' social support and self-efficacy. At the same time, organically integrate sports activities into mental health service plans to make exercise a regular solution for relieving stress and anxiety. In conjunction with corresponding health promotion, guide students to actively regulate their emotions and improve their psychological state through physical exercise. Eventually, a healthy atmosphere of “less screen viewing and more participation in activities” will be formed on campus, gradually reducing students' psychological dependence on smart phones.

### 4.5 Research limitation

Several limitations should be considered in future research. First, the results are based on students from various universities in Shandong, China, which may limit the representativeness and generalizability of the findings. Future studies should focus on larger, more diverse samples to further validate the results. Second, the cross-sectional design of this study does not allow for conclusions about causal relationships, highlighting the need for longitudinal and experimental research to confirm these findings. Finally, while this study identified the mediating role of social anxiety and the moderating role of physical activity in the relationship between perceived stress and problematic smartphone use, it is likely that other factors also play a role. Future research could explore these mechanisms from additional perspectives.

This study mainly focuses on individual psychosocial mechanisms, and future research can be extended to the environmental level. The estrangement of modern life from nature may prompt people to evade coping with real pressure through digital means, which is closely related to the decline of ecological intelligence. Subsequent studies can compare the intervention effects of “green exercise” and indoor activities on mobile phone addiction, which provides a new direction for integrating environmental psychology and behavioral addiction research.

## 5 Conclusion

The study reveals that perceived stress is a significant predictor of problematic smartphone use among college students. Social anxiety influences this behavior both directly and indirectly by affecting perceived stress. Additionally, physical activity plays a key moderating role in the relationship between perceived stress and problematic smartphone use, offering valuable insights into how physical activity impacts this issue. These findings highlight the importance of integrating physical activity into interventions and prevention strategies aimed at reducing problematic smartphone use among college students.

## Data Availability

The raw data supporting the conclusions of this article will be made available by the authors, without undue reservation.

## References

[1] Liu Q-X Fang X-Y Wan J-J Zhou Z-K. Need satisfaction and adolescent pathological internet use: Comparison of satisfaction perceived online and offline. Comput Human Behav. (2016) 55:695–700. 10.1016/j.chb.2015.09.048

[2] Loleska S Pop-Jordanova N. Is smartphone addiction in the younger population a public health problem? Prilozi. (2021) 42:29–36. 10.2478/prilozi-2021-0032 35032372

[3] Billieux J Maurage P Lopez-Fernandez O Kuss DJ Griffiths MD. Can Disordered Mobile Phone Use Be Considered a Behavioral Addiction? An update on current evidence and a comprehensive model for future research. Curr Addict Rep. (2015) 2:156–62. 10.1007/s40429-015-0054-y

[4] Brand M Young KS Laier C. Prefrontal control and internet addiction: a theoretical model and review of neuropsychological and neuroimaging findings. Front Human Neurosci. (2014) 8:375. 10.3389/fnhum.2014.00375 24904393 PMC4034340

[5] Rochat L Cruz GV Aboujaoude E Courtois R Brahim FB Khan R. Problematic smartphone use in a representative sample of US adults: Prevalence and predictors. Addict Behav. (2024) 162:108228. 10.1016/j.addbeh.2024.108228 39700606

[6] Witkiewitz K Bowen S Donovan DM. Moderating effects of a craving intervention on the relation between negative mood and heavy drinking following treatment for alcohol dependence. J Consult Clin Psychol. (2011) 79:54. 10.1037/a0022282 21261434 PMC3157314

[7] Askarizadeh G Poormirzaei M Hajmohammadi R. Identity processing styles and cell phone addiction: The mediating role of religious coping. Tehran: Research in Religion and Health (2017).

[8] Zencirci SA Aygar H Göktaş S Önsüz MF Alaiye M Metintaş S. Evaluation of smartphone addiction and related factors among university students. Int J Res Med Sci. (2018) 6, 2210–6. 10.18203/2320-6012.ijrms20182805

[9] Hong L Lai X Xu D Zhang W Wu B Yu X. Distinct patterns of problematic smartphone use and related factors in Chinese college students. BMC Psychiatry. (2022) 22:1–9. 10.1186/s12888-022-04395-z 36451113 PMC9710163

[10] Billieux J Linden MVD Rochat L. The role of impulsivity in actual and problematic use of the mobile phone. Appl Cogn Psychol. (2010) 22:1195–210. 10.1002/acp.1429

[11] Botella L. Human Change Processes: The Scientific Foundations of Psychotherapy. New York, NY: Basic Books (1993).

[12] Gong Z Wang L Wang H. Perceived stress and internet addiction among chinese college students: mediating effect of procrastination and moderating effect of flow. Front Psychol. (2021) 12:632461. 10.3389/fpsyg.2021.632461 34262501 PMC8273309

[13] Song WJ Park JW. The influence of stress on internet addiction: mediating effects of self-control and mindfulness. Int J Ment Health Addict. (2019). 10.1007/s11469-019-0051-9

[14] Beranuy M Oberst U Carbonell X Chamarro A. Problematic Internet and mobile phone use and clinical symptoms in college students: the role of emotional intelligence. Comput Human Behav. (2009) 25:1182–7. 10.1016/j.chb.2009.03.001

[15] Li J Zhang X Liu F Pang L Sun Y Chen Z. Rethinking the core symptom of schizophrenia: an exploration of disorganized thought centrality in psychosis. Schizophr Res. (2025) 281:201–8. 10.1016/j.schres.2025.05.010 40393115

[16] Yuan X Li X Pang L Kang Y Hei G Zhang X. Association between Purpureocillium, amino acid metabolism and cognitive function in drug-naïve, first-episode schizophrenia. BMC Psychiatry. (2025) 25:524. 10.1186/s12888-025-06965-3 40405167 PMC12100923

[17] Cohen LH McGowan J Fooskas S Rose S. Positive life events and social support and the relationship between life stress and psychological disorder. Am J Community Psychol. (1984) 12:567–87. 10.1007/BF00897213 6496413

[18] Hye-Jin K Jin-Young M Kyoung-Bok M Tae-Jin L Seunghyun Y Hajo Z. Relationship among family environment, self-control, friendship quality, and adolescents' smartphone addiction in South Korea: findings from nationwide data. PLoS ONE. (2018) 13:e0190896. 10.1371/journal.pone.0190896 29401496 PMC5798771

[19] Seo DG Park Y Kim MK Park J. Mobile phone dependency and its impacts on adolescents' social and academic behaviors. Comput Human Behav. (2016) 63:282–92. 10.1016/j.chb.2016.05.026

[20] Alvi T Kumar D Tabak BA. Social anxiety and behavioral assessments of social cognition: a systematic review. J Affect Disord. (2022) 311:17–30. 10.1016/j.jad.2022.04.130 35490878 PMC9754122

[21] Lee CYS Goldstein SE. Loneliness, stress, and social support in young adulthood: does the source of support matter? J Youth Adolesc. (2015) 45:568–80. 10.1007/s10964-015-0395-9 26602564

[22] Elhai JD Levine JC Dvorak RD Hall BJ. Problematic smartphone use: A conceptual overview and systematic review of relations with anxiety and depression psychopathology. J Affect Disord. (2017) 207:251–9. 10.1016/j.jad.2016.08.030 27736736

[23] Lee-Won RJ Herzog L Park SG. Hooked on facebook: the role of social anxiety and need for social assurance in problematic use of Facebook. CyberPsychol Behav Soc Network. (2015) 18:567–74. 10.1089/cyber.2015.0002 26383178

[24] Lei J Russell A I. Have a fear of negative evaluation, get me out of here! examining latent constructs of social anxiety and autistic traits in neurotypical and autistic young people. J Autism Dev Disord. (2021) 51:1729–47. 10.1007/s10803-020-04657-3 32808152 PMC8084828

[25] Heimberg RG Brozovich FA Rapee RM. A cognitive-behavioral model of social anxiety disorder: update and extension. 2014. 10.1016/B978-0-12-394427-6.00024-8

[26] Magalhaes M. The effect of various physical exercise modes on perceived psychological stress. S Afr J Sports Med. (2016) 26:104. 10.17159/2413-3108/2014/v26i4a501

[27] Cai P Wang J Ye P Feng X Yang G Huang C. Physical exercise/sports ameliorate the internet addiction from college students during the pandemic of COVID-19 in China. Front Public Health. (2024) 11: 1310213. 10.3389/fpubh.2023.1310213 38179571 PMC10764417

[28] Lepp A Barkley JE Karpinski AC. The relationship between cell phone use, academic performance, anxiety, and satisfaction with life in college students. Comput Human Behav. (2014) 31:343–50. 10.1016/j.chb.2013.10.049

[29] Yang G Tan GX Li YX Liu HY Wang ST. Physical exercise decreases the mobile phone dependence of university students in China: the mediating role of self-control. Int J Environ Res Public Health. (2019) 16:4098. 10.3390/ijerph16214098 31652978 PMC6862431

[30] Ke Y Liu X Xu X He B Wang J Zuo L. Self-esteem mediates the relationship between physical activity and smartphone addiction of Chinese college students: a cross-sectional study. Front Psychol. (2023) 14:1256743. 10.3389/fpsyg.2023.1256743 38250119 PMC10797096

[31] Agnew RA. Foundation for a general strain theory of crime and delinquency^*^. Criminology. (2010) 30:47–88. 10.1111/j.1745-9125.1992.tb01093.x

[32] Mishalani RG Ji Y Mccord MR. Effect of onboard survey sample size on estimation of transit bus route passenger origin-destination flow matrix using automatic passenger counter data. Transport Res Record J Transport Res Board. (2011) 2246:64-73. 10.3141/2246-09

[33] Manyi J Simao X. The Effect of Different Physical Exercises on Mobile Phone Dependence of College Students. Paris: Atlantis Press (2019).

[34] Cohen S Kamarck T Mermelstein R A. global measure of perceived stress. J Health Soc Behav. (1983) 24:385–96. 10.2307/2136404 6668417

[35] Leary MR. Social anxiousness: the construct and its measurement. J Pers Assess. (1983) 47:66–75. 10.1207/s15327752jpa4701_8 6834234

[36] Peng C. The adaptation of interaction anxiousness scale in Chinese undergraduate students. Chin Mental Health J. (2004) 18:39–41. 10.3321/j.issn:1000-6729.2004.01.014

[37] Fu H-Y Wang J Hu J-X. Influence of physical education on anxiety, depression, and self-esteem among college students. World J Psychiatry. (2023) 13:1121. 10.5498/wjp.v13.i12.1121 38186731 PMC10768485

[38] Tamayo JPM Rocchi MA St-Denis B Bonneville L Beaudry SG A. motivational approach to understanding problematic smartphone use and negative outcomes in university students. Addict Behav. (2024) 148:8. 10.1016/j.addbeh.2023.107842 37778235

[39] Lai C. Problematic smartphone usage in singaporean university students: an analysis of self-reported versus objectively measured smartphone usage patterns. Healthcare. (2023) 11:3033. 10.3390/healthcare11233033 38063601 PMC10705925

[40] Peng Y Zhou H Zhang B Mao H Hu R Jiang H. Perceived stress and Mobile phone addiction among college students during the 2019 coronavirus disease: The mediating roles of rumination and the moderating role of self-control. Pers Individ Dif. (2021) 179:111222. 10.1016/j.paid.2021.111222 34429562 PMC8376708

[41] Zhao C Xu H Lai X Yang X Tu X Ding N. Effects of online social support and perceived social support on the relationship between perceived stress and problematic smartphone usage among chinese undergraduates. Psychol Res Behav Manag. (2021) 14:549–58. 10.2147/PRBM.S302551 33976576 PMC8106527

[42] Lee HS. Convergent study of the effect of university students' addiction to smartphones on self-esteem and self-efficacy: stress level and mental health as mediating factors. Korea Converg Soc. (2017) 8:139–47. 10.15207/JKCS.2017.8.1.139

[43] Herrero J Torres A Arenas E Urue A. Examining the empirical links between digital social pressure, personality, psychological distress, social support, users' residential living conditions, and smartphone addiction. Soc Sci Comp Rev. (2021) 39:1234–51. 10.1177/0894439321998357

[44] Wang W Mehmood A Li P Yang Z Niu J Chu H. Perceived stress and smartphone addiction in medical college students: the mediating role of negative emotions and the moderating role of psychological capital. Front Psychol. (2021) 12:660234. 10.3389/fpsyg.2021.660234 34366978 PMC8336678

[45] Crowe SF. Assessing the neurocognitive disorders of the diagnostic and statistical manual of mental disorders (fifth edition). Austr Psychol. (2015) 50:54–9. 10.1111/ap.12104

[46] Kandemir M. Predictors of Academic Procrastination: Coping with Stress, Internet Addiction and Academic Motivation. World Appl Sci J. (2014) 32:930–8. 10.5829/idosi.wasj.2014.32.05.60

[47] Chou WP Ko CH Kaufman EA Crowell SE Hsiao RC Wang PW. Association of stress coping strategies with internet addiction in college students: the moderating effect of depression. Compr Psychiatry. (2015) 62:1–8. 10.1016/j.comppsych.2015.06.004 26343464

[48] Wang J Liu X Xu X Wang H Yang G. The effect of physical activity on sleep quality among chinese college students: the chain mediating role of stress and smartphone addiction during the COVID-19 pandemic. Psychol Res Behav Manag. (2024) 17:2135–47. 10.2147/PRBM.S462794 38826679 PMC11143986

[49] Garland EL Howard MO. Mindfulness-based treatment of addiction: current state of the field and envisioning the next wave of research. Addict Sci Clin Pract. (2018) 13:14. 10.1186/s13722-018-0115-3 29669599 PMC5907295

[50] Choi Y Lee H Lee S. Testing of cognitive-behavioral model of pathological internet use. In: The Proceedings of the Annual Convention of the Japanese Psychological Association. Tokyo: Japanese Psychological Association (2008).

[51] Borchers LR Gifuni AJ Ho TC Kirshenbaum JS Gotlib IH. Threat- and reward-related brain circuitry, perceived stress, and anxiety in adolescents during the COVID-19 pandemic: a longitudinal investigation. Soc Cogn Affect Neurosci. (2024) 19:nsae040. 10.1093/scan/nsae040 38874967 PMC11219304

[52] Ling Y Huebner ES Liu J Liu WL Zhang J Xiao J. The origins of hope in adolescence: a test of a social–cognitive model. Pers Individ Diff. (2015) 87:307–11. 10.1016/j.paid.2015.08.016

[53] Korniienko IO Barchi BV. The relationship between problematic use of smartphones and social anxiety. J Intellect Disabil Diag Treat. (2020) 8:133–41. 10.6000/2292-2598.2020.08.02.7

[54] Heimberg R Brozovich FA Rapee R. A cognitive behavioral model of social anxiety disorder: update and extension. In: Clinical Handbook of Psychological Disorders: A Step-by-Step Treatment Manual (4th Edn.). Amsterdam: Elsevier (2010).

[55] Mattick RP Clarke JC. Development and validation of measures of social phobia scrutiny fear and social interaction anxiety. Behav Res Ther. (1998) 36:455–70. 10.1016/S0005-7967(97)10031-6 9670605

[56] Li X Li W Liu M Xiao W Zhou H. How does shyness affect Chinese college students' tendency to mobile phone addiction? Testing the mediating roles of social anxiety and self-control. Front Public Health. (2022) 10:902425. 10.3389/fpubh.2022.902425 35910898 PMC9326250

[57] Zuo L Wang Y Wang J Yao Y Yang G. Physical activity influences negative emotion among college students in China: the mediating and moderating role of psychological resilience. Healthcare. (2025) 13:1170. 10.3390/healthcare13101170 40428006 PMC12110846

[58] Yang G Li Y Liu S Liu C Jia C Wang S. Physical activity influences the mobile phone addiction among Chinese undergraduates: the moderating effect of exercise type. J Behav Addict. (2021) 10:799–810. 10.1556/2006.2021.00059 34546969 PMC8997213

[59] Meng-Jiao LI Jie C Xin-Ying LI. Genetic and Neurobiological Mechanisms of Non-drug Addictions. Adv Psychol Sci. (2012) 20:1623–32. 10.3724/SP.J.1042.2012.01623

[60] Seabrook EM Kern ML Rickard NS. Social networking sites, depression, and anxiety: a systematic review. Jmir Mental Health. (2016) 3:e50. 10.2196/mental.5842 27881357 PMC5143470

[61] Wang J Xu X Wu Q Zhou C Yang G. The mediating effect of subject wellbeing between physical activity and the internet addiction of college students in China during the COVID-19 pandemic: a cross-sectional study. Front Public Health. (2024) 12:1368199. 10.3389/fpubh.2024.1368199 38645442 PMC11026853

[62] Wang J Li L Wu Q Zhang N Shangguan R Yang G. Effects of parental psychological control on mobile phone addiction among college students: the mediation of loneliness and the moderation of physical activity. BMC Psychol. (2025) 13:60. 10.1186/s40359-025-02385-w 39838486 PMC11749423

[63] Chris E. The strength model of self-control in sport and exercise psychology. Front Psychol. (2016) 7:314. 10.3389/fpsyg.2016.00314 26973590 PMC4773600

[64] Yang G Shangguan R Ke Y Wang S. The influence of acute aerobic exercise on craving degree for university students with mobile phone dependency: a randomized controlled trial. Int J Environ Res Public Health. (2022) 19:8983. 10.3390/ijerph19158983 35897357 PMC9331807

[65] Hayes AF. PROCESS: A Versatile Computational Tool for Observed Variable Mediation, Moderation, and Conditional Process Modeling (2012). Available online at: http://www.afhayes.com/public/process2012.pdf

